# Personalized Smart Clothing Design Based on Multimodal Visual Data Detection

**DOI:** 10.1155/2022/4440652

**Published:** 2022-03-24

**Authors:** Haijuan Deng, Minglong Liu

**Affiliations:** ^1^School of Art and Design,Suzhou University, Suzhou 234000, China; ^2^School of Physical Education,Suzhou University, Suzhou 234000, China

## Abstract

In the traditional clothing customization system, only the designer participates in the clothing design, and the style is single. In the face of numerous styles, the user just repeatedly arranges and combines the styles, but does not realize the user's innovative design. In this paper, we propose a novel multitask deep convolutional neural network training method for task-by-task transfer learning, and learn deep image features for image retrieval tasks on noisy user click data. Image retrieval model based on image-text multimodal correlation features: this paper uses image-text multimodal correlation features to calculate the correlation between query keywords and images, and calculates the correlation between images and images. In this paper, the method of automatic generation of clothing style is researched, and parameterized coding is designed for it. Taking the typical style of suits as the initial population, through the human-computer interaction interface, the scoring value is assigned to the fitness value to carry out the evolution process. The binary string of the suit style generated by the genetic algorithm is decoded through the decoding algorithm and rules, decoded into a visual style diagram of the style of the suit style, and the style diagram of the suit style is automatically drawn. From the perspective of clothing design, this paper summarizes the general design methods of outdoor sports smart clothing, follows the integration of people-clothing-environment, and proposes a wearer-centered design concept to deeply explore the rationality of outdoor sports smart clothing design methods. This paper further solves the key problems in the design process, and takes the fashion of clothing as the core principle to design the structure of clothing modeling. The fabric selection of suits is based on the principle of clothing comfort. The key is to realize the outdoor sports monitoring function of suits through sensing technology. The design uses Arduino as an electronic prototype platform, so as to detect the heart rate of the human body during exercise and the microclimate temperature under the clothes. This kind of suit with monitoring function is ultimately a combination of sensing device and clothing. It not only has monitoring function, but also has the aesthetic concept of clothing design and conforms to the performance of human body structure, which will provide reference and reference for the design of outdoor sports smart clothing.

## 1. Introduction

Based on the new environment of big data and algorithm technology revolution, all kinds of commodities cover the consumer market at an extremely fast speed and refresh people's lives on the premise of meeting the potential needs of contemporary people [1]. With a “color revolution,” the clothing changed the gray-green tone, which rescued the Chinese people from the aesthetic fatigue of clothing and brought a new fashion to the society. Today, with the rapid expansion of the global knowledge economy, one after another technological innovation is leading another revolution in the field of clothing [2]. Smart clothing, which was originally a cutting-edge field, began to emerge as early as the 20th century when the styles were diverse. With the unique function different from traditional clothing and the personal advantage that other carriers and media are incomparable, along with the vigorous development of the electronic information technology industry and the extensive research and application of interdisciplinary research in various fields, it has become a high-profile industry in the industry [3]. With the sweeping wave of wearables, smart clothing not only brings unprecedented convenience and speed to people's daily life, but also increasingly impacts people's beautiful vision for future cities. Its entry trend has spawned active thinking in the global clothing capital market, and has attracted forward-looking technology-based companies to set foot in the field of smart clothing to cooperate in the development of innovative technological textiles [4, 5].

With the increasing market coverage, the industry has gradually realized the importance of intelligent clothing design theory, and after solving the basic wearing performance problems, began to think about how to use the fashion power of the letter of guarantee in clothing to deal with what is happening in the industry [6]. Design alienation is a major subject in the current design research field. Therefore, it is necessary for us to return to the original intention of smart clothing design to conduct in-depth analysis and discussion, so as to promote the harmonious and orderly coordination of social achievements in clothing, which is a very modern carrier [7, 8]. While continuously improving the technical level, we have an philosophical and pragmatic attitude towards the industry, skillfully incorporate flexible humanistic relationships into the design consideration process, and bring the flexible and unquantifiable things in clothing back to the language of the rapid entry of technology into the market. In this case, it should be organically combined with the development of intelligent new technologies, put forward innovative and reasonable design patterns, and convey people-oriented service design concepts to meet people's actual needs. Therefore, it is necessary to give a critical warning. It is intended to provide reference operation experience and development direction for the new design concept of smart clothing.

The image retrieval model based on pretrained feature representation uses large-scale text corpus and annotated image data to train text feature representation and image feature representation respectively, and then map the text feature space and image feature space to the same feature space after linear transformation based on canonical association analysis. The feature space calculates the similarity. The image retrieval model based on multitask training uses a multitask learning framework to learn image feature representations suitable for image retrieval tasks by means of task-by-task transfer training. Based on the multimodal correlation feature representation, the similarity is calculated using the image feature representation and text feature representation in the same continuous space, and the retrieved images are sorted by comprehensively considering the similarity between the images in the retrieval results. Specifically, the technical contributions of this paper can be summarized as follows.

First, we analyze the research status and methods of interactive evolutionary computing, and conduct genetic algorithm automatic design research on customized clothing. Taking suit as an example, the coding and decoding scheme designed by the interactive genetic algorithm and the algorithm flow of the interactive genetic algorithm are introduced. Through the coding scheme, the genetic operation of the genetic algorithm can be carried out, and through the decoding algorithm, the binary string of the offspring of the suit style generated by the genetic algorithm can be decoded and the corresponding suit style diagram can be automatically drawn.

Second, in order to ensure the safety, health and comfort of the wearer in the optimized design, and realize the intelligent health monitoring function on the clothing, the more important issues are the connection of electronic components and the integration of system hardware. According to the selected sensor placement position and the trend of the structure dividing line, the research and design of this subject carry out the concealment of the conductive lines and the adjustment of the process. Compared with ordinary suits, the garments have added moldings for arranging the wires. The optimized design of the clothing prototype innovatively uses conductive sewing thread to connect the LED and the control board together, and summarizes the matters needing attention during the sewing process.

Third, the research design of this subject overcomes the flexibility and persistence of the interconnection between the sensor and the executive circuit board through optimized design. The electronic components must be integrated into the unobtrusive parts of the clothing without hindering the movement of the body. The ability to disassemble to facilitate laundry is the three most common and important obstacle issues. Secondly, this paper evaluates the design method and prototype wearing experience. An evaluation scale was set up, and 20 relevant personnel respectively evaluated and scored the “wearer-centered 3F + 1I” design mode and the wearing experience of the clothing prototype, which were centered on the research of this topic, and obtained the evaluation degree of various aspects of the ready-to-wear.

## 2. Related Work

The style-aided design system developed by related scholars using genetic programming technology encodes a series of relevant sizes of styles into chromosomes, and the system evolves styles according to the user's choice [9]. Because some domain knowledge of style design is not considered in the coding, the generated style is generated. Most styles are not practical. Relevant scholars proposed an interactive genetic algorithm, that is, to obtain a fitness function through interaction with the user, so as to solve the evaluation problem of style fitness, but requiring the user to give a fitness function, so that the style types of the system are limited [10, 11]. This kind of research is a theoretical research on the intelligent deformation of the flat style renderings, which cannot be related to the actual clothing design and production [12].

To realize intelligent garment CAD, establishing a mathematical model of garment structure design is the foundation. In the aspect of optimizing the mathematical model of clothing structure design, there are many experts who study it [13]. Through the collection and analysis of experimental data, the researchers established a mathematical model of the design of the jacket model by using the method of inductive statistics, which provided a technical basis for the intelligent production of clothing [1]. Relevant scholars calculated stepwise regression through C++ language, and established a mathematical model of clothing structure design between 6 control parts and other 31 detail parts [14]. In view of the introduction of the feature table technology in the development and design of clothing templates by some current clothing computer-aided design systems, relevant scholars have studied the model parameter analysis method, the explicit relationship between the coordinates and parameters of the control point of the template geometry, and constructed a reusable template geometry [15]. The model expounds the basic principles of model object feature extraction. Although the model variant design based on parameters is realized, it is only oriented to the graphic features of the model, and is not combined with customer needs and the accumulation of previous design knowledge and experience [16].

Through the drawing experiment of a series of patterns and the analysis of related data, the researchers determined the key data of the deformed sub-basic pattern-the proportional relationship between the concave amount of the armhole arc and other related data, thus establishing loose clothing [17]. Related scholars put forward a production concept that realizes product deformation and customization through flexibility and rapid response [18]. This concept can not only meet the individual needs of customers, but also does not affect the efficiency of enterprises. The working principle of this system is that when an individual size is input into the system, the system will retrieve the most similar version according to the body size, and then intelligently modify the version.

Relevant scholars pointed out that in recent years, many countries have begun to adopt large-scale multi-expert collaborative systems, multiple knowledge representations, comprehensive knowledge bases, self-organizing problem-solving mechanisms, multi-disciplinary collaborative problem-solving and parallel reasoning, expert system tools and environments, and artificial neural networks [19]. The latest artificial intelligence technologies such as knowledge acquisition and learning mechanism are used to realize the fourth-generation expert system with multiple knowledge bases and multiple agents. The long-term goal of expert systems is to explore the basic principles of human intelligence and machine intelligence, and to study the use of automata to simulate human thinking processes and intelligent behavior. This goal goes well beyond computer science and touches almost all disciplines in the natural and social sciences. Therefore, the current forecast for the development of expert systems is focused on the near term goal of building expert systems of the type that can be used to replace high-level human mental work.

Relevant scholars have constructed the structure of the clothing style design expert system, analyzed the key issues of realizing the intelligent human-computer interaction interface, and established the style knowledge representation model and style reasoning mechanism [20]. Because clothing design belongs to a kind of creative thinking, and clothing itself is a kind of flexible body, its design mostly depends on experience and inspiration, and design knowledge is difficult to quantify and regularize [21].

## 3. Methods

### 3.1. Analysis of Cross-Modal Image Retrieval Task

The cross-modal image retrieval task is to give a user query text keyword and return the corresponding image. In order to learn the text features and image features suitable for cross-modal image retrieval, this paper uses the user click data of commercial search engines as weakly supervised data to learn the feature representation of different modalities of text and images. The cross-modal image retrieval task only uses the image and query keyword information and user click information in the retrieval model training, and does not use any textual description information about the image in the web page. When retrieving model tests, only images and query keyword information are used.

The testing process requires that a query keyword and several images related to the keyword are given, and these images are sorted so that the images most relevant to the query keyword are ranked first, and the irrelevant images are ranked last. Finally, the ranking results are evaluated using the NDCG evaluation annotation. N DCG is defined as follows:

(1) $$\begin{array}{c} NDCG@d=Z_{d}•\prod_{j=0}^{d-1}\frac{\log\left(j-1\right)}{2^{r^{j}}-1}. \end{array}$$

*Z*_*d*_ is the normalization factor such that ideally the size of NDCG@d is 1. The performance of the final model is evaluated in terms of the average NDCG@d value of all query keywords in the test set. According to the official instructions of the MSR-Bing image competition, NDCG@25 is used as the evaluation index of the final performance of the image retrieval model, and the results of NDCG@25 are normalized with *Z*_*d*_ = 0.0178.

### 3.2. Image Retrieval Model Based on Pretrained Feature Representation

#### 3.2.1. Text Feature Learning

**Figure 1:**
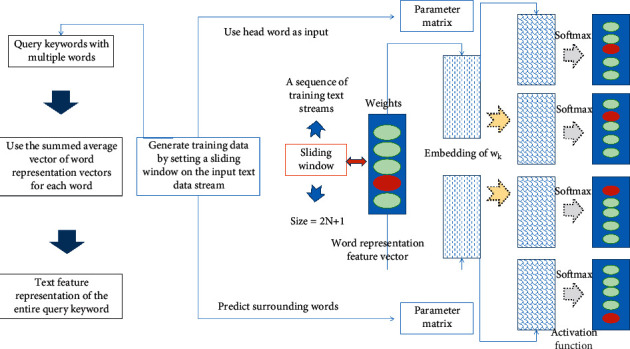
Schematic diagram of skip-gram model structure.

The core idea of Skip-gram is based on the assumption that similar words should have similar contexts. As shown in Figure 1, in the skip-gram model, the training data is generated by setting a sliding window in the input text data stream. In a sliding window at each moment, the surrounding words are predicted using the center word as input. Specifically, input words are represented as 1-of-V sparse vectors, where V is the size of the entire vocabulary in the training data, and each word in the vocabulary is represented as a length V with only one dimension of 1, the others are sparse vectors of 0. In the forward propagation process, the input word is firstly mapped to the word representation space in the form of a vector through the word embedding parameter matrix M. Then through a series of nonlinear changes, another parameter matrix M′ is mapped into a new vector, which is mapped back to a 1-of-V sparse vector representation of all surrounding words through a softmax activation function. During backpropagation, prediction errors are propagated back to the network to update the two parameter matrices. After the training process converges, the parameter matrix *M* is treated as the word representation feature vector of the corresponding word.

Specifically, given a sequence of training text streams *w*1, *w*2, *w*3, ..., *w*_*K*_, the training objective of the skip-gram model is to maximize the following expected probability:

(2) $$\begin{array}{c} L=\frac{1}{K+1}\prod_{k=0}^{K-1}\log_{p}\left(w_{k-j}|w_{k};-N<j<N\right). \end{array}$$

Among them, *w*_*k*_ is the center word, *w*_*k*+*j*_ is the surrounding word, and *N* represents the actual sliding window size is 2*N* + 1. Conditional probabilities are expressed in the form of the following softmax function:

(3) $$\begin{array}{c} p\left(w_{k+j}|w_{k}\right)=\frac{\exp\left(v^{\prime }w^{T}v_{w_{k}}\right)}{\prod_{w=0}^{V-1}\exp\left(v_{w_{k}}w^{T}\right)}. \end{array}$$

Among them, *v*_*w*_ and *v*_*w*_′ are the input and output of the hidden variable *M*′. Since the size of *V* is generally very large, negative sample sampling is usually used in actual training to approximate the above algorithm.

In image retrieval tasks, since the query keyword is usually not a single word, for query keywords with multiple words, the summed average vector of the word representation vectors of each word is usually used as the text feature representation of the entire query keyword.

#### 3.2.2. Image Feature Learning

Different from traditional image feature representation, the visual features learned by deep neural networks trained on supervised image recognition tasks have more high-level semantic information and are more suitable for tasks such as image recognition and image retrieval. Given a picture and a trained deep network model for image recognition and classification, usually the output of a certain layer in the pretrained neural network after inputting the picture is used as the feature representation of the image, and the layer closer to the output layer of the neural network is used. The output feature representation is closer to high-level semantic information. Conversely, the feature representation of the output of the network layer that is closer to the image input layer is closer to the underlying image feature representation (color, local contour, etc.).

In order to verify the advantages of deep image features over traditional image features, this paper compares the classification performance of SVMs represented by different image features on the PASCAL VOC image recognition task. The Pascal VOC 2017 object classification task dataset contains a total of 10,000 images in 20 categories including man-made and natural objects. Since objects in the images of this dataset are generally not centered, in general, the Pascal VOC 2017 object recognition task is considered more challenging than ILSVRC-2020.

In image retrieval tasks, since query keywords generally correspond to semantic-level text feature representations, depth image features are more suitable for keyword-based image retrieval tasks than traditional image features without high-level semantic information.

#### 3.2.3. Image Retrieval Model Based on Canonical Association Analysis

After obtaining the text feature representation and image feature representation, in order to solve the difficulty in the open image retrieval task—the query keywords are diverse and cover a wide range, it is necessary to have a method that can directly analyze the relationship between the text feature representation and the image feature representation. The most intuitive solution is to map the text feature representation and image feature representation into another latent space at the same time.

Canonical Correlation Analysis (CCA) can construct a latent space that can evaluate the features of two different spaces through statistical-based mathematical transformation. Here this paper uses user click data as supervision information for one-to-one mapping between image feature representations and text feature representations. User click data can be defined as (*T, I*), for each *I*_*i*_ ∈ *I*, there is a corresponding query keyword *q*_*i*_ ∈ *T* that the user has clicked on the image. Where *I*_*i*_ and *q*_*i*_ are the *i*-th image and query keyword of the image set *I* and the query keyword set *T*, respectively, and *T* ∈ *R*_*n*_ × *dq*, *I* ∈ *R*_*n*_×*d*_*I*_, *d*_*I*_ is the dimension of the image feature, *n* is the number of query key-image pairs. The role of CCA is to find two linear transformations *w*_*T*_ and *w*_*I*_, so that the correlation coefficient between *T* and *I* after the linear transformation is the largest. The linear transformation process of *T* and *I* is as follows:

(4) $$\begin{array}{c} T_{W_{T}}=\left(\begin{array}{ccc} w_{T}^{I}v_{q_{1}} & \begin{array}{cc} w_{T}^{I}v_{q_{2}} & \cdots \end{array} & w_{T}^{I}v_{qn} \end{array}\right),\\ I_{W_{I}}=\left(\begin{array}{ccc} w_{T}^{I}v_{I_{1}} & \begin{array}{cc} w_{T}^{I}v_{I_{2}} & \cdots \end{array} & w_{T}^{I}v_{In} \end{array}\right). \end{array}$$

The optimization goal of the CCA algorithm is to maximize the correlation *ρ* between *T*_*wT*_ and *I*_*wI*_:

(5) $$\begin{array}{c} p=\underset{w_{T},w_{I}}{Max}corr\left(I_{W_{I}},T_{W_{T}}\right). \end{array}$$

For the above optimization goal, *I* and *T* can be standardized once and then solved by SVD, and then sorted according to the eigenvalues from large to small, several singular values or eigenvalues can be obtained as the correlation coefficient of the data. *w*_*T*_ and *w*_*I*_ corresponding to these different correlation coefficients constitute a matrix.

### 3.3. Image Retrieval Model Based on Multitask Training and Image Retrieval Model Based on Multimodal Correlation Features

#### 3.3.1. Image Retrieval Model Based on Multitask Training

The performance of image feature representation is most important for this type of task. In general, to learn feature representations directly from search engine image data, each query keyword can be viewed as a separate category.

The parameters of the entire neural network are divided into two parts, one part is the parameter Ws shared between the neural networks corresponding to different query keywords at the bottom layer, and the other part is the exclusive parameter *Wqi* of the neural network corresponding to each query keyword. The task-by-task transfer training is inspired by the fact that the feature representation of each layer of the deep neural network is different, and the higher the layer, the more abstract the feature representation. The mutual sharing of parameters between the neural networks corresponding to each query keyword accelerates the training speed, and at the same time ensures that some query keywords with fewer training samples can also be effectively trained.

#### 3.3.2. Image Retrieval Model Based on Multimodal Correlation Features

Since the correlation between images and texts can be directly calculated in the multimodal correlation feature space, a natural idea is to build an image retrieval engine based on multimodal key features. However, another very important requirement for building an image search engine is to sort the returned images, and rank the images that are frequently clicked by users and the images that are most relevant to the query keywords.

The Bag-of-Words similarity based ranking method (BoWDNN-R) model is calculated between the bag-of-words representation vector directly based on the query keyword and the bag-of-words representation vector obtained after the image is passed through the deep convolutional neural network model based on the bag of words representation. Similarity. Given an image I and a query keyword *q*, the bag of words of the query keyword is denoted as Bq, and the bag of words corresponding to the image I is denoted as *f*(*I*_*a*_)*E*. The similarity between the two can be calculated by the following formula:

(6) $$\begin{array}{c} SIM\left(I,q\right)=\frac{B_{q}•Ef\left(I\right)}{\left\|B_{q}•E\right\|\left\|f\left(I\right)\right\|}. \end{array}$$

Considering that the user click data has recorded a certain degree of preference of the user to find the content, generally speaking, the more similar the number of images in a user clicked image to other clicked images, the closer the image is to the user's expectations. Search results. That is to say, the relative similarity between the two images in the candidate images clicked by the user can also be used as an important reference for ranking the search results. Here, this paper adopts a modified PageRank model (MPM) to measure the similarity between the image and the query keyword by the similarity between the image and the image:

(7) $$\begin{array}{c} SIM\left(I,q\right)=\frac{1}{N-1}\prod_{j=0}^{N-1}sim\left(I,I_{j}\right)=\frac{1}{N-1}\prod_{j=0}^{N-1}sim\left[\begin{array}{cc} f\left(I_{a}\right) & f\left(I_{b}\right) \end{array}\right]. \end{array}$$

Among them, *I*_*j*_ is one of the *N* images most similar to image *I* in the user clicked image. In order to avoid the influence of noisy images, only the most similar 5 images are taken for calculation, namely *N* = 5.

### 3.4. Parametric Coding Design of Clothing Style

According to the research on suits, it can be seen that daily suits have two forms: flat lapel collar and lapped lapel collar. Therefore, this paper takes the flat lapel collar as the research object to analyze the collar shape of the suit. Before binary coding the parameterization of the collar of the suit, it is necessary to understand the collar structure of the suit.

The binary codes corresponding to the 8 parameters of collar depth, collar position, lapel width, collar opening depth, angle between lapel angle and fold line, collar angle, upper collar width, and placket width are connected from high to low. The binary encoding scheme of the suit collar parameters is shown in Table 1.

There are ten representative samples of suit style styles, that is, there are 10 typical styles and styles of suits, which are: youthful vitality, mature and steady, showing off and perverted, reserved, masculine, feminine, business occupation, leisure and natural, heroic and unrestrained, simple and honest. The eight parameters corresponding to the collar type of the above typical style suits are binary coded according to the method in Table 1, and the binary code corresponding to the collar can be obtained.

The outer contour style of the suit is relatively monotonous. The main factors that control the change of the outer contour of the suit are the shape of the hem and the self-cultivation of the waist. In this paper, 6 bit binary coding is used to control the change of the outer contour of the suit. Among them, for the hem, 4 bit binary code is used for control; for waist circumference, 8 bit binary code is used for control. The presence or absence of the decorative pocket square of the suit is controlled by a 1 bit binary code. The parametric binary coding scheme of suit profile is shown in Table 2.

### 3.5. Automatic Generation of Suit Styles Based on Interactive Genetic Algorithm

Taking the top of a suit as a chromosome, the styling elements of the suit style are coded separately. There are 11 elements of suit modeling, which are collar depth, collar position, lapel width, collar opening depth, angle between lapel corner and fold line, collar angle, collar width, placket width, Hem, pocket square, waist. Each modeling element is encoded in binary with different digits. *A* is the depth of the collar, *B* is the position of the collar, *C* is the width of the lapel, *D* is the opening depth of the collar, *E* is the angle between the lapel corner and the fold line, *F* is the angle of the collar, *G* is the width of the collar, H is the placket width, I is the hem, *J* is the pocket square, and K is the waist. The number of coded digits depends on the value range of the style parameters of the suit.

In the interactive design module, the clothing style scheme and score selected by the user are used as the input of the interactive genetic algorithm, and 10 typical styles are assigned to the initial population, that is, the number of the initial population is 10.

In this section, the genetic algorithm is designed to maximize the user's rating of clothing; the depth of the collar, the position of the collar, the width of the lapel, the depth of the collar opening, the angle between the lapel angle and the fold line, the angle of the collar, and the width of the collar width, access width, hem, pocket square, and waist circumference are the parameters required for 8 collar designs as decision variables.

The mutation operator can increase the diversity of the population and prevent the population from falling into local optimum. When the individual offspring of the population satisfies the condition of mutation probability, one dimension of the individual gene segment of the offspring is randomly initialized within its value range.

**Figure 2:**
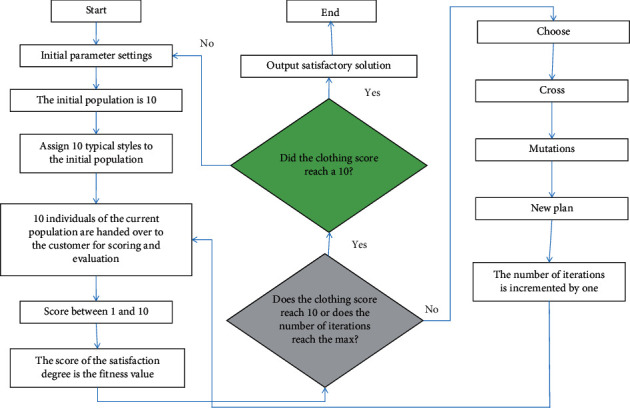
The flow chart of the optimal design of the suit collar genetic algorithm.

The population of the clothing evaluation algorithm process based on genetic algorithm is generated by random initialization. In order to improve the evaluation efficiency, a variety of classic clothing styles are introduced as the initial population to help users who blindly choose clothing to quickly generate their favorite clothing matching plans. The clothing evaluation process based on genetic algorithm is shown in Figure 2.

The specific operation is to let the user select two or more preferred options among the alternatives and rate them. Individuals are randomly selected to crossover to generate new individuals in the chosen scheme until the population size is satisfied and two individuals are generated per cross.

In addition, individuals that do not satisfy the constraints will be crossed again until the interruption condition is satisfied. Then a mutation operator is introduced to process the population in one step. If the above constraints are not met it will mutate again until the break condition is met.

### 3.6. Binary Decoding of Suit Style

The purpose of the binary decoding algorithm is to decode the binary string of the offspring of the suit style style generated by TS-IGA, decode it into a visual style diagram of the suit style style, and automatically draw the corresponding suit style style diagram. According to the descendant binary string generated by the genetic algorithm, the corresponding collar type parameters are calculated, and the style diagram of the corresponding suit collar type is drawn, which is divided into three steps:

(1) According to the binary descendant solution generated in the genetic algorithm, the corresponding parameter values of the suit collar can be obtained; (2) according to the obtained parameter values of the suit collar, the style diagram of the suit collar is drawn; (3) during the drawing process, the coding rules for suit collars are written to eliminate illogical solutions.

In the descendant population generated by the interactive genetic algorithm, there will be some that do not meet the suit customization rules and need to be eliminated, and some suit decoding rules need to be set to eliminate unreasonable solutions. The rules for decoding a suit collar are as follows:

(1) In the process of decoding the collar shape of a suit, it is stipulated that the position of the collar must be smaller than the collar depth *c*; (2) The lapel width *d* must be smaller than the collar depth *c*; (3) The collar opening depth *L* must be smaller than the lapel width *d*; (4) The position of the lapel corner shall not exceed the curve of the shoulder.

## 4. Results and Analysis

### 4.1. Simulation Verification and Clothing Prototype Wearing Experience Analysis

In the simulation experiment, the data monitored by the sensor is transmitted to the fusion center, and the local judgment results of each sensor are normalized in the fusion center, and the normalized results form a judgment matrix. Then the synthetic operation result adopts the method of maximum membership degree to obtain a comprehensive judgment on the human somatosensory.

**Figure 3:**
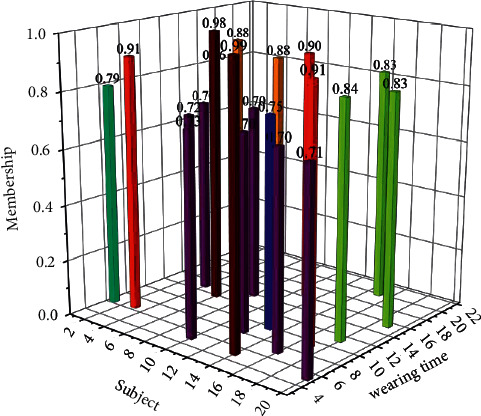
Membership of monitoring results.

The experiment consisted of 20 subjects, uniformly dressed, in the same laboratory environment, prescribed the same time period, and performed simulated exercise respectively. After exercising, record and choose your overall feeling of physical comfort based on your own experience. During the test, the speed of the pedals was fixed at 35 km/h (the pedal speed was maintained through a tachometer to provide feedback to the subjects). After wearing for 30 minutes, the experiment obtained the normalized judgment results of each sensor and the subjective overall feeling of the 20 groups of subjects at a certain moment after wearing. The membership degree of monitoring results is shown in Figure 3.

**Figure 4:**
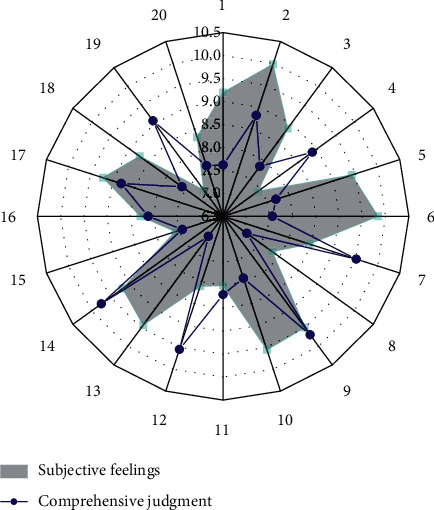
Comparison of sensory evaluation.

Due to the different physical constitutions of the 20 subjects, the sensitivity of body sensations, and the various uncontrollable self-factors such as different exercise intensity that can be tolerated, after the simulated exercise in the same time period, there will be differences in the degree of judgment. Through the simulation experiment, the corresponding reliability evaluation of the research design of this subject can be made objectively. From Figure 4, it can be concluded that the subjective feelings of the subjects and the comprehensive judgment of the intelligent algorithm are basically consistent, indicating that the research design and application of this subject is reasonable, reliable to a certain extent, and has practical value.

The functional design of smart clothing is similar to the functional design of traditional clothing, and it is a design process centered on the wearing user. Based on the wearer's wearing experience, how the wearer evaluates the smart clothing from the subjective point of view, the usability of the smart clothing can be subjectively evaluated from four main aspects, namely psychology, physiology, function and management.

Psychological evaluation is mainly based on the wearing effect of the wearer, such as color matching, style structure, and whether it fits. Generally, it is not only required to look good, but also required not to interfere with the normal work of the wearing user. On the other hand, safety psychology refers to the wearer's consideration of the safety of smart clothing, mainly considering whether the system clothing operates reliably and will not affect the health of the human body.

The physical properties of electronic components have been the most concerned issue in the research on the usability of smart clothing in the past ten years. It directly or indirectly affects the usability of clothing, such as causing physical discomfort, restricting movement, and damaging the body. This can be regarded as an important requirement for the usability of clothing, the main issues such as weight and volume of electronic components, distribution location, firmness, etc., are based on the understanding of human physiological evaluation.

Function is an attribute of electronic equipment to function on clothing carrier. For example, how to monitor the health signs of wearing users. Previously, the core of the function of smart clothing was the development of integration with wearable technology, and now researchers are more focused on optimizing the function to improve the overall usability of the clothing.

Functionality can be considered in terms of manufacturability, data transfer rate, battery life, etc.; management must consider the daily care of smart clothes, from the perspective of clothing and electronics, whether it is easy to maintain, whether it is washable, durability, etc.

Therefore, we evaluate the usability of wearing tops with outdoor monitoring function designed in this subject, and then conduct investigation and analysis through subjects.

### 4.2. Evaluation of Personalized Smart Clothing Design

**Figure 5:**
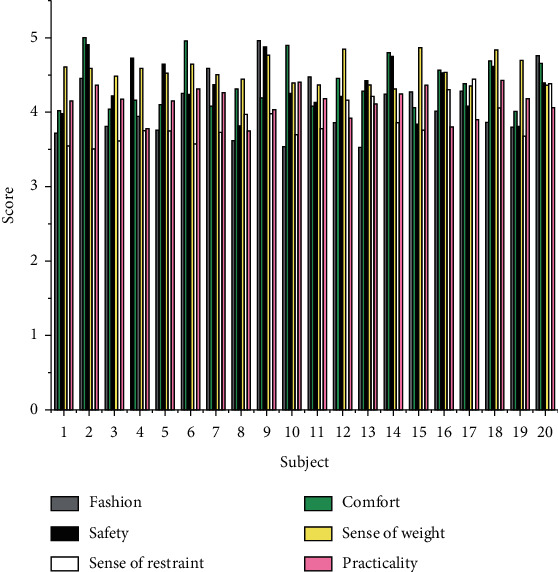
Scores for each evaluation direction.

**Figure 6:**
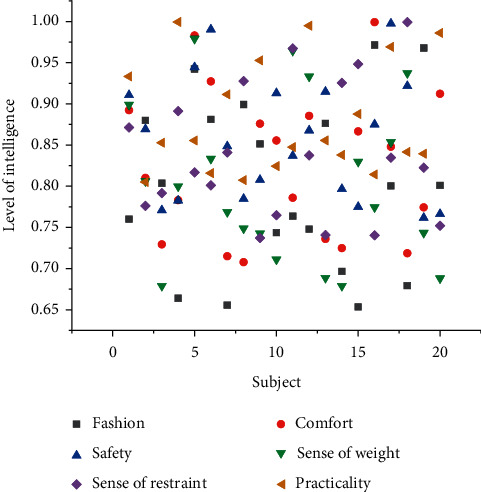
The intelligence level of each evaluation direction.

According to the evaluation direction of smart clothing, the questionnaires were investigated from four aspects: psychological feeling evaluation, physiological feeling evaluation, functional experience evaluation, and management evaluation, mainly including the following contents: fashion, comfort, safety, dress weight, restraint, practicality, data transfer speed. We use appropriate language to summarize and express the subjective scale for evaluating clothing. The scale is divided into different evaluation levels. The numerical value marked at the endpoint of each level represents a degree. We select the corresponding number to represent the evaluation direction of the clothing. Attitude (the larger the value, the greater the appraiser's recognition or degree of the content of the evaluation direction). There are five grades on the scale, which are respectively weak, weak, medium, strong, and strong. These five descriptive words correspond to each other one by one according to the subjective feelings and give the evaluation according to the numerical value on the scale. After the questionnaires were collected, simple mathematical processing was performed on the questionnaires using SPSS software, and the data obtained are shown in Figures 5 and 6.

The fashion intensity of this research design is between 4 and 5, belonging to the category of “strong” to “strong.” This shows that the prototype aesthetic design of the clothing is good, the color matching is reasonable and attractive, it can meet the psychological needs of the wearer, and the style and shape conform to the characteristics of sports. The scores of comfort strength and fashion strength are similar and belong to the same category, and the weight feeling is close to 3, indicating that a slight weight feeling can be felt due to the electronic components, but it does not hinder the smooth progress of the exercise.

Due to the low score of restraint, it belongs to the category of “weak” to “weak,” and the restraint brought by clothing to the human body and electronic components to the human body can be ignored, indicating that the design is ergonomic, the fabric is smooth and elastic, and it fits well. By simulating the exercise process, the clothing shows good safety performance, and the safety strength falls into the category of “strong” to “strong”; the practical strength is close to “strong,” indicating that the intelligent solution operates stably and can achieve the expected health monitoring. Electronic components can be disassembled to meet the requirements of washing.

In this part, 20 relevant personnel were invited to evaluate the research design of this subject. In order to make the evaluation method simple and easy to implement, this paper adopts the form of questionnaire survey. The content of the questionnaire mainly includes two parts: one is the evaluation of the general methods and application methods of clothing prototype design; the other is the evaluation of the wearing performance and upper body effect of the clothing prototype through wearing experience.

**Figure 7:**
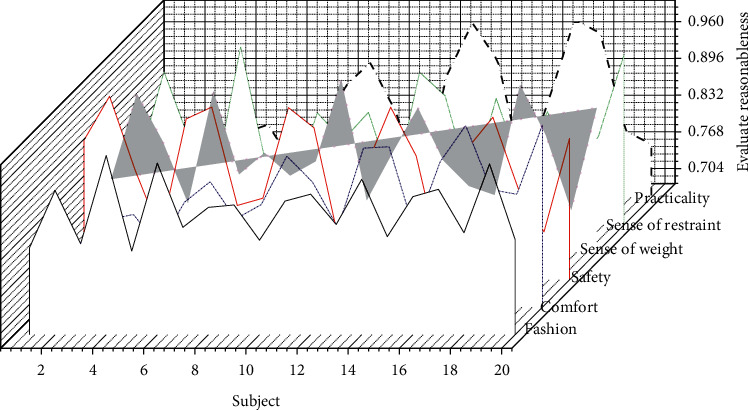
Statistics for evaluating reasonableness.

**Figure 8:**
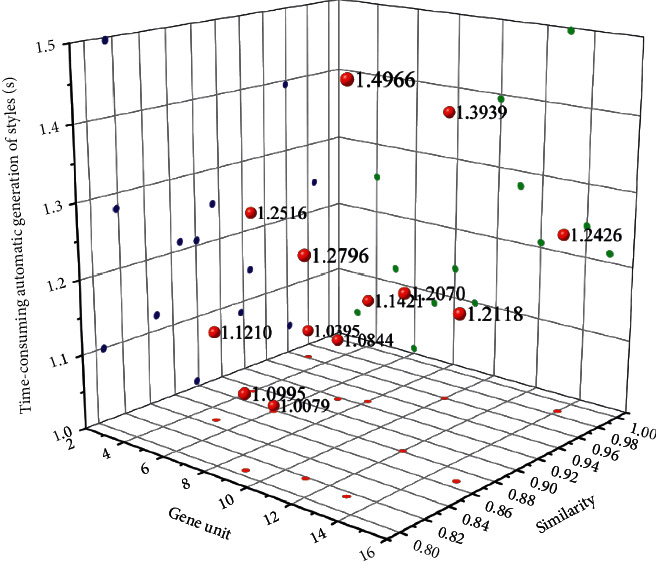
Time-consuming automatic generation of suit styles based on interactive genetic algorithm.

While designing and applying this research, in the form of a questionnaire, the relevant personnel are invited to discuss the rationality of the design model of “wearer-centered 3*F* + 1*I*” based on the general design method of smart clothing in this research. The overall application evaluation is given. The statistic for evaluating rationality is shown in Figure 7. The time-consuming of automatic generation of suit styles based on interactive genetic algorithm is shown in Figure 8.

The research design of this subject adopts the “wearer-centered 3*F* + 1*I*” smart clothing design model, and the relevant personnel evaluate the rationality of its use and the overall design application based on the suit with monitoring function as the prototype as “reasonable,” which shows that this mode has certain practical guiding significance and reference value in design and application. In addition, we summarize the advantages given by relevant personnel on the research of this topic: by sorting out and summarizing the design methods and design theories of general smart clothing, and then proposing the “wearer-centered 3*F*+1*I*” smart clothing design mode, the overall structure is simple and easy. It can effectively guide the follow-up researchers' research methods, is practical, and can help expand design ideas to a certain extent. Using the survey data as a reference for specific operations will make the design rigid and lack auxiliary application expressions. At the same time, some suggestions are also put forward: combine the general methods of design based on design patterns and summary design, analyze specific situations in detail, truly focus on the wearer, think in multiple dimensions and directions, use flexibly, and not rigid dogma. The database of clothing applications can involve more advanced technologies and application examples in multiple fields.

## 5. Conclusion

Based on the characteristic that there is a certain correlation between image information and text information, this paper proposes a new neural network structure, and innovatively uses search engine user click data as weakly supervised data to learn image-text correlation features. A common feature space that can represent image information and text information at the same time is obtained. In this feature space, not only can image-image, text-text feature correlations be calculated directly, but also cross-modal feature correlations such as image-text, text-image can be directly calculated. Different from the traditional image feature representation based on image recognition training on artificially labeled datasets, the image feature representation and text feature representation learned based on the method in this paper are more in line with the distribution of real user needs. According to the automatic design method of suit style by interactive genetic algorithm, this paper carries out parametric binary coding design on the design elements of suit style. The method of interactive genetic algorithm based on typical style is used to optimize the inheritance of clothing, and carry out benign evolution of suit design according to the user's evaluation. It is innovatively designed by using the genetic operation of crossover and mutation of the genetic algorithm, and is decoded into a visual style diagram of the style of the suit style through the decoding algorithm, and the style diagram of the suit style is automatically drawn. The research design of this subject takes western clothing with monitoring function as the main object. From clothing design to technical design, the fuzzy comprehensive decision-making of multi-sensor fusion is used to effectively comprehensively judge the human comfort level under real-time monitoring. The suit with this monitoring function is based on clothing, which effectively combines electronic components with clothing. The research design of this subject is optimized through structural adjustment and technological innovation, which basically solves the flexibility and durability of the interconnection between the sensor and the execution circuit board. The design and application of the research of this topic realizes the real-time monitoring function of outdoor sports, and combines the important physiological characteristics of the human body to comprehensively judge the physical state during exercise.

## Figures and Tables

**Table 1:** Binary encoding scheme of suit collar parameters.

Suit collar parameters	Gene unit	Genotype	Cumulative phenotype
Collar deep	4 bit unit	0010	1001001000000000
Neck width	6 bit unit	0001	1101111000000011
Neckline angle	4 bit unit	0011	1101111000110000
Angle between the lapel corner and the fold line	3 bit unit	0000	0011100000000000
Neck opening depth	8 bit unit	0100	0010111000000000
Lapel width	5 bit unit	0111	0001111000011100
Neckline position	6 bit unit	0101	0110011000000010

**Table 2:** Parametric binary coding scheme of suit profile.

Suit profile parameters	Unit	Representing meaning	Genotype (sample)	Phenotype
*Waistline*	6 bit unit	1	Loose	Round, no pocket square
		0	Slim fit	Round, with pocket square

*Hem*	4 bit unit	0	Straight	No pocket square, loose
		1	Round	No pocket squares, slim fit

*Pocket square*	8 bit unit	1	Tradition	Flat, pocket square, slim fit
		0	Fashion	Flat, pocket square, loose

## Data Availability

The data used to support the findings of this study are available from the corresponding author upon request.
